# State-dependent changes in peak alpha frequency during visual engagement in children with and without autism spectrum disorder

**DOI:** 10.3389/fpsyt.2025.1634384

**Published:** 2025-10-06

**Authors:** Masuhiko Sano, Tetsu Hirosawa, Daiki Soma, Masafumi Kameya, Keigo Yuasa, Mai Yasumoto, Yoko Osaka, Yuko Yoshimura, Yuka Shiota, Sanae Tanaka, Chiaki Hasegawa, Mitsuru Kikuchi

**Affiliations:** ^1^ Department of Psychiatry and Neurobiology, Graduate School of Medical Science, Kanazawa University, Kanazawa, Japan; ^2^ Department of Child and Adolescent Psychiatry, Graduate School of Medical Science, Kanazawa University, Kanazawa, Japan; ^3^ Research Center for Child Mental Development, Kanazawa University, Kanazawa, Japan

**Keywords:** autism spectrum disorder, peak alpha frequency, magnetoencephalography, neurodevelopment, brain regions

## Abstract

Peak alpha frequency (PAF) is a neurophysiological marker of cortical maturation and cognitive function. We aimed to examine PAF reactivity to a visually engaging eyes-open (EO) condition, during which children watched a muted preferred video, compared to a dark-room (DR) resting state without sound, in children with ASD and their TD peers. We analyzed magnetoencephalography data from 68 cortical sources in children aged 5–10 (ASD: n=22; TD: n=29), calculating PAF during a resting-state DR condition and an EO condition involving silent video viewing. Linear mixed-effects models were used to assess the effects of diagnosis, condition, and their interaction on PAF, controlling for age and sex. The results indicated a significant interaction between diagnosis and condition in the right temporal region, where TD children consistently showed a higher PAF in the EO condition relative to the DR condition, whereas children with ASD did not. Furthermore, in TD children, greater PAF reduction in the right temporal region correlated with lower social responsiveness scores, suggesting a link between PAF reactivity and social functioning. These findings suggest that atypical PAF modulation in response to sensory input may reflect altered neural mechanisms underlying social information processing in ASD. Understanding PAF reactivity patterns can inform the development of ASD biomarkers.

## 1 Introduction

Autism spectrum disorder (ASD) is a neurodevelopmental condition characterized by social and communicative challenges, as well as restricted, repetitive behaviors and highly focused interests (1). Although these features commonly arise during early childhood, they tend to persist throughout life. With the rise in ASD rates and its substantial socioeconomic implications (2), timely and accurate diagnosis has become increasingly critical. However, identifying ASD can be complex owing to subtle behavioral cues, limited clinical assessment time, and frequently co-occurring conditions such as anxiety or hyperactivity. These complications underscore the importance of examining biological and physiological markers of ASD to improve diagnostic precision. In recent years, neuroimaging has emerged as an essential tool for advancing our understanding of the neural mechanisms underlying ASD (3).

Neuroimaging methods have offered new insights into the biological mechanisms of ASD, with electroencephalography (EEG) and magnetoencephalography (MEG) being particularly advantageous. By capturing the electrical and magnetic signals of the brain without generating noise or radiation, EEG and MEG are especially well-suited for pediatric populations. Alpha oscillations (8–12 Hz)—the dominant resting-state rhythm—are of special interest in ASD research. Alpha activity is most pronounced with eyes closed and typically decreases in amplitude once the eyes are open and visual input is introduced (4). Although alpha rhythms have been implicated in social coordination (5, 6) and broader information processing across thalamocortical and cortico-cortical pathways (7–10), studies on alpha “power” in ASD have shown inconsistent results, ranging from lower (11–13) or higher (14) alpha power to no observable group differences (15, 16). These discrepancies underscore the limitations of relying solely on alpha power as a marker for atypical brain function in ASD.

Recent meta-analyses and systematic reviews have indicated the critical influence of recording condition—namely, eyes-open (EO) or eyes-closed (EC)—on resting-state EEG findings in ASD (17). Alpha suppression—the characteristic reduction in alpha amplitude upon eye opening—may be diminished in individuals with ASD, resulting in relatively higher alpha power in the EO condition, compared with typically developing (TD) participants, while showing minimal group differences in the EC condition (18). These observations underscore the need to distinguish between these two states when probing for atypical brain oscillations. Moreover, investigating peak alpha frequency (PAF), as opposed to focusing exclusively on alpha power, may yield clearer insights into the neurophysiological mechanisms underlying ASD, especially when examining the transition from EC to EO.

PAF—the resting-state alpha oscillation at which power is maximal—has garnered considerable attention for its clinical and developmental significance. It correlates with various cognitive abilities in TD individuals. For example, a higher PAF is associated with more efficient cognitive functioning, including better working memory, faster processing speed (19–21), and higher intelligence quotient (IQ) scores (22). PAF also undergoes significant age-related changes, making it a valuable indicator of brain maturation in TD populations (23, 24). Specifically, PAF shifts from approximately 8 to 9 Hz in younger children (aged 5–7 years) to an ‘adult-like’ rhythm of 10–12 Hz by mid-adolescence (25, 26). Consequently, PAF is a sensitive measure of alpha oscillatory development (27) and, in some cases, considered more informative than alpha power alone (28). Given the fundamental role of alpha rhythms in numerous brain processes, investigating PAF in neurodevelopmental disorders such as ASD remains both relevant and promising.

Numerous EEG and MEG studies have been conducted to explore differences in PAF between younger individuals with ASD and their TD peers (14, 16, 29–39). Among studies focusing on resting-state EC condition, findings suggest that children with ASD without intellectual disability exhibit higher PAF around 7 years of age (29), with this pattern persisting until about 10 years of age (32), yet reversing at around 13–14 years of age (38). In contrast, an EO EEG study involving visual stimulation revealed that TD children around the age of 7 years had a higher PAF than those with ASD (33). Methodological variability—including differences in imaging modality (EEG (33, 38) vs. MEG (29, 32)), methods of PAF calculation (visual inspection (29, 32) vs. automated approaches (16, 33)), and participant characteristics—complicates direct comparisons across these findings. Additionally, several studies showed no significant differences in PAF between ASD and TD groups in EC (14, 16, 35) or EO condition, whether with robust visual stimulation (39) or minimal stimulation, such as a fixation cross (37). These mixed results highlight the potential role of recording conditions (EO vs. EC) in modulating observed differences in PAF. However, no previous research has directly compared the transition between EO and EC conditions within the same sample of children, a gap that the current study aims to fill.

Several studies have consistently shown a positive association between PAF and nonverbal IQ (NVIQ) across both ASD and TD populations (29–34, 38), across EC (29, 30, 32, 38) and EO conditions (with minimal or robust visual stimulation) (31, 33, 34). However, the relationship between PAF and autistic traits, such as social difficulties, remains controversial. Chung et al. (39) reported that a lower PAF at 12 months predicted more frequent repetitive and restricted behaviors at 24 months in children with ASD in EO condition involving robust visual stimulation. Similarly, Kameya et al. (37) found that lower PAF was associated with more pronounced autistic traits measured using the Social Responsiveness Scale (SRS) (40) in TD children around 7 years of age in EO condition involving minimal visual stimulation, although this association was not significant within the ASD subgroup. Conversely, several studies have shown no significant associations: Edgar et al. (32) found no relationship between SRS-measured autistic traits and PAF in children aged approximately 10 years in the EC condition, and Finn et al. (38) reported no significant link among older children (approximately 13–14 years old) in a similar condition. Additionally, Dickinson et al. (30) observed no significant correlation between SRS-measured autistic traits and PAF in adult populations in the EC condition. Interestingly, in their non-ASD subgroup, higher autistic symptom severity (quantified by the Autism Diagnostic Observation Schedule; ADOS) was negatively correlated with PAF, a pattern not observed in the ASD group. These mixed and sometimes contradictory findings highlight the complex relationship between PAF and autistic traits. They also underscore the importance of considering participant age, task condition (EO versus EC), and measurement instrument when examining how PAF might reflect or mediate core features of ASD.

While alpha activity is most prominent in posterior regions, recent findings suggest that alpha oscillations in nonposterior areas also carry meaningful developmental and cognitive information, particularly in the context of neurodevelopmental disorders. For example, Dickinson et al. (33) demonstrated that the relationship between PAF and both age and non-verbal IQ differs across regions and diagnostic groups: age was positively associated with frontal, central, and occipital PAFs only in TD children, whereas non-verbal IQ was associated with frontal and central but not occipital PAFs only in children with ASD. In a longitudinal study, Dickinson et al. (34) further showed that frontal and central PAFs at 24 months predicted verbal developmental quotient at 36 months, whereas occipital PAF did not. More recently, Kameya et al. (37) found that right temporal PAF was associated with autistic traits only in TD children and that age–PAF associations in the cingulate regions differed between the TD group and the group with ASD. Collectively, these findings underscore that alpha oscillations outside classical posterior regions may reflect distinct neurodevelopmental mechanisms, justifying a whole-brain approach to PAF analysis.

Despite growing evidence that PAF may capture neural processes relevant to ASD, no study has systematically examined how PAF changes between resting-state and robust visual-input conditions within a single pediatric cohort. Given that alpha oscillations are particularly sensitive to visual stimulation and that atypical alpha suppression occurs in ASD (17, 18), examining shifts in PAF between these two states may yield new insights into the neurophysiological underpinnings of ASD. We hypothesized that the degree of PAF change from a dark-room (DR) resting condition without sound to an EO condition involving silent video viewing would differ between children with ASD and their TD peers. Moreover, because PAF has been linked to both cognitive functioning and autistic traits in young children (29, 33, 37), we further hypothesize that this change is associated with social-communicative difficulties. In the present study, we aimed to investigate (a) whether the change in PAF between a resting-state DR condition and an EO condition with robust visual stimulation (but no auditory input) differs between children with ASD and their TD peers, and (b) whether this difference correlates with SRS-measured autistic traits. Focusing on this fundamental transition in brain reactivity, we aimed to deepen our understanding of how visual input modulates alpha oscillations in neurodevelopmental disorders.

While regional variation in PAF is well established, we did not aim to analyze region-specific differences in this study. Our prior work using a similar cohort and analytic pipeline (37) showed that although PAFs varied across anatomical regions, the spatial distribution of PAF was broadly comparable between children with ASD and their TD peers. Based on those findings, we aimed to focus the current investigation on condition- and group-level effects rather than regional differences.

## 2 Methods

### 2.1 Study design and participants

In this prospective observational study, we recruited children aged 5–10 years with ASD, along with their TD peers, to obtain MEG recordings. The ASD group comprised 23 children recruited from Kanazawa University and its affiliated hospitals. ASD diagnoses were made according to the Diagnostic and Statistical Manual of Mental Disorders, Fourth Edition (DSM-IV) criteria (41) and confirmed by experienced psychiatrists and psychologists using the Diagnostic Interview for Social and Communication Disorders (DISCO) (42) or the second edition of the ADOS (ADOS-2) (43, 44). The control group included 30 TD children with no reported behavioral or language difficulties.

Children were excluded if they had blindness and/or deafness, had other neuropsychiatric disorders, or were using medication. Children with known intellectual disabilities were also excluded. Family history of ASD was not screened for in the control group. Written informed consent was obtained from the parents of each participant before enrollment. The procedures were approved by the Ethics Committee of Kanazawa University Hospital and were conducted in accordance with the Declaration of Helsinki. This research is part of the Bambi Plan at the Kanazawa University Research Center for Child Mental Development (https://kodomokokoro.w3.kanazawa-u.ac.jp/en/). Although some participants were included from our previous research (37, 45), there was no overlap in results, as the objectives of the previous studies differ substantially from those of the present work.

From the initial sample, one boy with ASD and one TD girl were excluded because they were unable to complete the MEG or psychological assessments described below.

### 2.2 MEG recording

MEG data were recorded using a 151-channel Superconducting Quantum Interference Device (SQUID) whole-head coaxial gradiometer system (PQ 1151R; Yokogawa/KIT, Kanazawa, Japan), housed in a magnetically shielded room (Daido Steel Co., Ltd., Nagoya, Japan). This system was custom-designed with a child-sized MEG helmet to optimize sensor placement for smaller head sizes (46), which helps to limit head movement. All MEG signals were low-pass filtered at 500 Hz and sampled at 2,000 Hz.

Participants underwent MEG recordings under following two conditions: a resting-state DR condition and an EO video-viewing condition. In the DR condition, children lay supine in a darkened room while focusing on a centrally presented fixation cross, approximating a classical resting-state setup. In the EO condition, children remained in the same supine position while watching a silent video of their choice projected onto a screen. A selection of popular children’s video programs was made available, from which each participant selected a preferred video before recording. Although this individualized selection reduced standardization across participants, it was prioritized to minimize anxiety, enhance comfort, and reduce head movement during data acquisition.

To further encourage minimal head movements, a research staff member remained in the MEG room with each child during data collection. This strategy proved effective for most participants; however, one TD girl was unable to complete the recording owing to discomfort and difficulty staying still.

All MEG recordings were performed between 11:00 AM and 3:00 PM, and no child demonstrated overt signs of drowsiness based on visual inspection of the waveforms. Although a longer recording period is generally desirable in MEG studies, maintaining stillness in children, especially those with ASD, posed significant challenges. Given the challenge of keeping young children stationary, we set a minimum recording duration of 50 s, consistent with our previous studies (37, 45). Each condition (DR and EO) was recorded for 120 s to ensure that sufficient artifact-free data could be retained after removing segments affected by movement. This decision aligns with prior research suggesting that even 38 s of artifact-free EEG data can provide stable estimates of spontaneous measures, such as PAF (33, 47). As described in Sections 2.6 to 2.9, for each participant and each condition, the continuous MEG recording was segmented into 5-s epochs with 80% overlap, and power spectral density (PSD) was computed for each epoch. The PSDs were then averaged, and PAF was determined from the resulting mean spectrum. This approach reduces the influence of transient noise and enhances the robustness of the PAF estimation.

### 2.3 Assessment of intelligence and severity of autism symptoms

To evaluate intellectual functioning, we administered the Kaufman Assessment Battery for Children (K-ABC) (48) or its second edition (KABC-II) (49) to all participants, depending on their availability at the time of assessment. The K-ABC features a Mental Processing Scale (MPS) that measures problem-solving abilities through simultaneous and sequential processing tasks. The KABC-II features the Mental Processing Index (MPI), which serves a similar purpose by assessing general mental processing. Because our study focused on children with ASD without intellectual disabilities, we set a minimum score requirement of 70 on these scales, consistent with standard criteria distinguishing intellectual disability from average intellectual functioning (1). One boy with ASD was excluded from subsequent analyses for scoring below 70 in on the MPS (K-ABC).

Autistic traits were assessed using the SRS (40) or its second edition (SRS-2) (50), both completed by a parent. These instruments provide a continuous measure of social functioning, ranging from impaired to above average, with higher scores indicating more pronounced autistic traits. Because scores along a continuum can be obtained, these measures can help identify and understand individuals with milder ASD, as well as those with non-ASD conditions who also show social impairments (51).

### 2.4 Magnetic resonance imaging

Structural brain images were acquired using a 1.5 Tesla (T) magnetic resonance imaging (MRI) scanner (SIGNA Explorer; GE Healthcare, USA), with a T1-weighted gradient echo sequence employing the Silenz pulse sequence. Silenz is designed to reduce acoustic noise and shorten scan times (52, 53), making it particularly suitable for pediatric populations. Imaging parameters included: repetition time (TR) = 435.68 ms, echo time (TE) = 0.024 ms, flip angle = 7°, field of view (FOV) = 220 mm, matrix size = 256 × 256 pixels, and slice thickness = 1.7 mm, resulting in 130 transaxial images. Although the use of thicker slices and a lower matrix size leads to a slightly reduced spatial resolution compared with standard protocols, this setup offered adequate anatomical references while minimizing scanning duration to enhance participant compliance.

### 2.5 Co-registration of MEG data and MRI images

Co-registration of MEG data and MRI images was based on specific marker locations. Four distinct markers were identified on both MEG and MRI: the midline frontal point, vertex, and bilateral mastoid processes. Magnetic field-generating coils served as the markers for MEG, while lipid capsules acted as markers for MRI owing to their distinct appearance as high-intensity regions. Additionally, points on the mastoid processes, nasion, and skull surface were visually identified on MRI images. Typically, 15–25 points were marked for each participant to ensure accurate co-registration.

### 2.6 MEG data preprocessing

MEG data analyses were performed using Brainstorm (54), an open-source software tool freely available under the GNU General Public License. The preprocessing steps followed the guidelines of the Organization for Human Brain Mapping (55) and were identical to those used in our previous work (37, 45). First, the data were downsampled to 500 Hz, and noisy sensors were identified and excluded. Second, notch filters were applied at 60, 120, and 180 Hz to remove the power-supply noise. A band-pass filter (0.5–200 Hz) was then used to isolate the relevant frequency range. Third, independent component analysis was performed to identify and remove components related to blinks and cardiac artifacts. Fourth, segments with apparent motion artifacts or radio frequency interference were visually inspected and excluded by an author (D.S.), who was blinded to participant identities. Finally, the remaining data were segmented into continuous 5-second intervals, with each participant required to have at least 10 segments (i.e., 50 seconds of total data) for further analysis.

### 2.7 Atlas-guided source reconstruction and segmenting

Signal source estimation was performed using the original anatomy of each participant. An anatomically constrained MEG approach was employed to estimate brain signal sources by placing anatomical constraints on the estimated sources. Specifically, a head model was computed using the overlapping spheres algorithm (56) with the default source space, which is a lower-resolution cortical surface representation comprising 15,000 vertices. We used weighted minimum-norm estimation to determine source orientation constraints (57). An identity matrix was used as the noise covariance because no noise recordings were available. Signal sources were then grouped into 68 regions based on the Desikan–Killiany atlas (58), using principal component analysis.

### 2.8 Computing spectral power

Welch’s method was used to compute the spectral power of the 68 regions defined using the Desikan–Killiany brain atlas. The continuous time series was segmented into 5-s epochs with 80% overlap, and each segment was windowed using a Hamming window. PSD was computed for each epoch and then averaged to obtain a robust estimate of the power spectrum. The resulting spectra had an approximate frequency resolution of 0.2 Hz.

### 2.9 Measurement of peak alpha frequency

**Figure 1:**
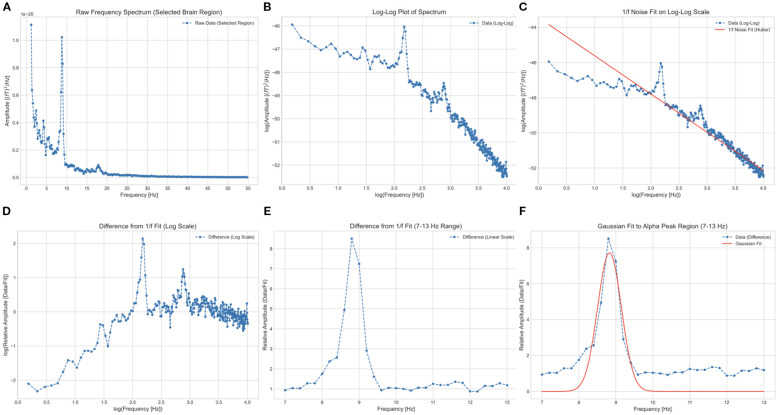
Illustration of the procedure for estimating peak alpha frequency from a single signal source localized to the left caudal anterior cingulate cortex. **(A)** Raw power spectral density in the 1–55 Hz range. The horizontal and vertical axes represent frequency and absolute power, respectively. **(B)** Logarithmic transformation of frequency and power values (blue dotted line). **(C)** Robust linear regression (Huber’s method, M = 1.35) used to model the 1/f component (red line) based on log-frequency. **(D)** Subtraction of the predicted 1/f component from the log-transformed data. **(E)** Exponentiation of power and frequency, with the alpha band restricted to 7–13 Hz. **(F)** Gaussian function fitting (red curve) applied to the residual in **(E)**. The frequency at the peak (vertex) of this curve was defined as the peak alpha frequency.

**Figure 1A** shows the power spectral density of a representative temporal source. The PAF for each of the 68 signal sources was calculated according to established protocols (33, 35), identical to the method used in our previous study (37).

A key challenge in identifying alpha peaks in EEG/MEG power spectra is the dominant 1/f trend (59), which can mask subtle alpha-band activity. To address this, we applied a log-transformation to both frequency and power in the 1–55 Hz range (**Figure 1B**), rendering the 1/f component as a linear function of log-frequency.

We then used robust linear regression (Huber’s method, M = 1.35) to predict the 1/f component of the log-transformed power based on log-frequency, as robust methods are more resilient to outliers than traditional least-squares methods (60, 61). The predicted 1/f component (red line in **Figure 1C**) was subsequently subtracted (**Figure 1D**). The residuals were exponentiated, and the alpha band between 7 and 13 Hz was isolated (**Figure 1E**).

These alpha-band spectra were fitted to a Gaussian curve using the least-squares method (62). The peak of the Gaussian curve was designated as the PAF (**Figure 1F**). Any peak outside the predefined 7–13 Hz alpha band was deemed absent and excluded from further analysis. This approach provides a clear PAF estimate, which is particularly advantageous for participants with multiple or indistinct alpha peaks.

**Figure 2:**
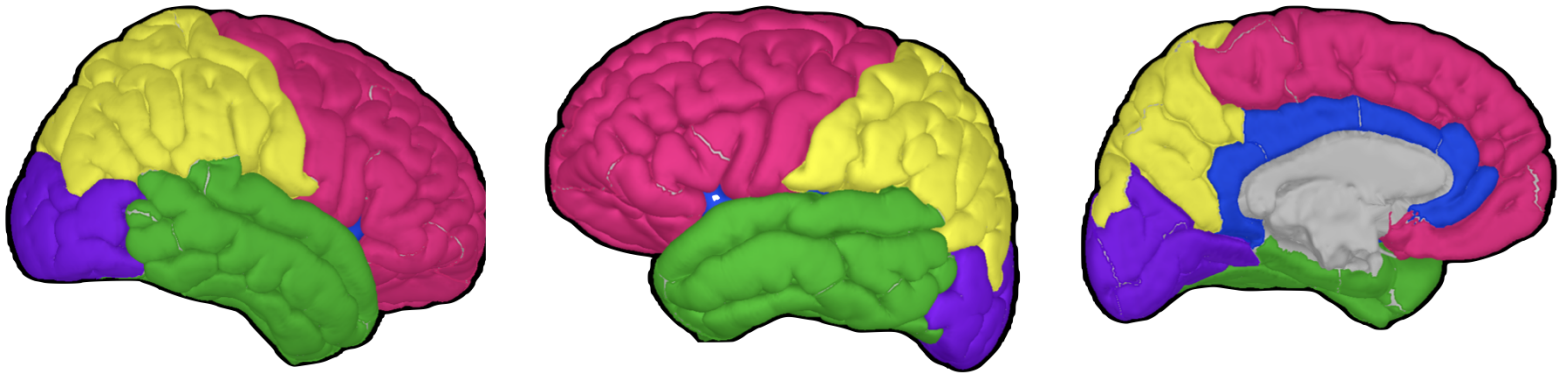
Brain regions for peak alpha frequency analysis.

PAF was first computed for each of the 68 regions in the Desikan–Killiany atlas. To improve stability, these 68 regions were subsequently grouped into 10 broader macro-regions: cingulate, frontal, occipital, parietal, and temporal regions in each hemisphere (58) (**Figure 2**). Within each macro-region, PAF values were averaged only across micro-regions that produced valid estimates within the 7–13 Hz range. If no valid estimates were available in a given macro-region, that macro-region was treated as missing. This yielded up to 10 representative PAF measures per participant.

All figures were generated using the Matplotlib library (63) in Python.

This figure depicts the major brain regions analyzed for peak alpha frequency, based on the Desikan–Killiany atlas. Three-dimensional brain models were generated using Brainstorm software (54) and the ICBM152 MRI atlas (64, 65). Left and middle panel: lateral view highlighting frontal (red), parietal (yellow), temporal (green), and occipital (purple) lobes. Right panel: medial view showing the cingulate cortex (blue) alongside other visible regions.

### 2.10 Statistical analysis

All statistical analyses were performed using Stata version 17.0 (StataCorp LLC, College Station, TX, USA). Group differences in age, K-ABC score, and SRS score between the ASD and TD groups were assessed using two-tailed Student’s t-tests, while sex differences were evaluated using chi-square tests.

The primary goal of this study was to determine whether the effect of experimental condition (DR vs. EO) on PAF differed between diagnostic groups (ASD vs. TD) across specific brain regions. We also examined whether the magnitude of PAF changes induced by the EO condition depended on baseline measures obtained under the DR condition.

To address these questions, we used linear mixed-effects models, with PAF in each region as the dependent variable. Fixed effects included diagnosis (ASD vs. TD), experimental condition (DR vs. EO), their interaction term, age, and sex. A random intercept for each participant accounted for within-subject correlations, capturing individual variability and the hierarchical structure of the data. Because the PAF values from corresponding regions in opposite hemispheres (e.g., left vs. right temporal lobes) may not be fully independent, we applied a Bonferroni correction for multiple comparisons across the five major regions of interest (cingulate, frontal, occipital, parietal, and temporal). Accordingly, statistical significance was set at p < 0.01 (0.05/5) (66).

If any model indicated a significant diagnosis-by-experimental condition interaction, further investigation was performed to evaluate the effect of experimental condition on PAF within each diagnostic group separately. For these subgroup analyses, linear mixed-effects models were used, including experimental condition (DR vs. EO) as a fixed effect, along with age and sex, and random intercepts were retained for each participant. After applying the Bonferroni correction for two comparisons, the threshold for statistical significance in these subgroup analyses was set at p < 0.025.

We then calculated the PAF change score (EO minus DR) and, for each region, ran separate linear regression models predicting this change from baseline PAF (DR), diagnosis, age, and sex. We additionally tested models including an interaction term between diagnosis and baseline PAF to assess group differences in the predictive relationship. The same Bonferroni-corrected threshold (*p* < 0.01) was used to determine statistical significance.

Lastly, we examined whether autistic traits predicted EO-induced changes in PAF, focusing only on the brain regions that showed a significant diagnosis-by-condition interaction in the mixed-effects model. Specifically, we calculated the difference in PAF between the two experimental conditions (EO minus DR) and conducted linear regression analyses predicting total SRS scores from this difference, age, and sex.

Before fitting these models, we verified that all relevant assumptions were satisfied, including normality of residuals, homoscedasticity, absence of multicollinearity, and appropriate distributions of random effects.

## 3 Results

### 3.1 Participants

The final sample included 22 children in the ASD group and 29 children in the TD group, with age ranges of 60–95 months and 60–92 months, respectively. No significant difference was observed between the groups in sex, age, K-ABC MPS score. However, a significant difference was observed in total SRS score (t (49) = −6.44, p < 0.0001). Depending on the time of recruitment, 32 participants (13 TD and 19 ASD) completed the K-ABC-I, while 19 participants (16 TD and 3 ASD) completed the K-ABC-II. An exploratory analysis of K-ABC-II MPI scores revealed a significant group difference (*t* (17) = −2.19, *p* = 0.043). These findings are summarized in **Table 1**. For the ASD group, the mean ADOS-2 social affect, restricted and repetitive behavior, total, and comparison scores were 6.6 (SD = 3.7), 2.3 (SD = 1.5), 8.9 (SD = 4.4), and 4.9 (SD = 2.3), respectively. To clarify data quality, we examined the number of usable epochs retained after artifact rejection. In the DR condition, TD children had significantly more usable epochs than children with ASD (TD: 99.7 ± 2.7; ASD: 90.2 ± 3.8; *t (49*) = 2.10, *p* = 0.04). In the EO condition, the difference between these two groups was not significant (TD: 112.7 ± 1.1; ASD: 115.0 ± 0.9; *t (49*) = –1.56, *p* = 0.12). To further assess the reliability of our data, we evaluated the proportion of PAF estimates that fell within the physiologically expected range (7–13 Hz), summarized for each macro-region and condition. For example, the value of 84.6% for the left frontal region in the EO condition of the TD group was calculated with a denominator of 11 micro-regions × 29 participants, and a numerator equal to the number of PAF estimates within the 7–13 Hz range among those observations. In the TD group, these proportions were generally high across macro-regions (range: 70.7–97.9%), indicating robust alpha peak detection. In contrast, the ASD group showed greater variability and lower proportions in several macro-regions (range: 42.5–69.0%). These results are summarized in **Supplementary Table 1** and likely reflect increased signal variability, artifacts, or atypical alpha topography in children with ASD. Notably, after aggregation into 10 macro-regions, valid PAF values were obtained for nearly all participants. Specifically, in the TD group, 28–29 of 29 participants contributed values across macro-regions in the DR condition and 25–29 of 29 in the EO condition. In the ASD group, 20–22 of 22 participants contributed values in both the DR and EO conditions. Here, the ranges (e.g., 28–29) indicate the minimum and maximum number of participants with valid values across the 10 macro-regions. To complement this analysis, we also examined the quality of spectral fitting across regions by calculating the mean R² values between the modeled and observed PSDs. As shown in **Supplementary Table 2**, R² values were generally moderate across both groups and conditions (range: 0.385–0.766), with some variability depending on brain region. To further assess the plausibility of the estimated PAFs, authors T.H. and M.S. independently conducted visual inspections of the PSDs and corresponding fitted curves across all regions. This step served as an additional quality check, ensuring that the estimated alpha peaks were consistent with the observed spectral profiles.

**Table 1:** Participant characteristics.

	TD	ASD	χ^2^ or t	*p*
N	29	22		
Sex (% male)†	55.2	63.6	0.37	0.54
Age (months)‡	73.6 ± 1.8	74.3 ± 2.1	0.29	0.78
Total SRS score‡	47.9 ± 1.7	68.9 ± 3.0	6.43	<0.001*
K-ABC MPS score‡	115.0 ± 3.5	103.1 ± 4.2	-2.02	0.052
K-ABC-II MPI scores‡	119.8 ± 4.1	97 ± 9.9	-2.19	0.043*
Number of Epochs, DR‡	99.7 ± 2.7	90.2 ± 3.8	2.10	0.04*
Number of Epochs, EO‡	112.7 ± 1.1	115.0 ± 0.9	-1.56	0.12

†Chi-square test.

‡Student’s t-test.

Numbers are presented as mean ± SD.

ASD, autism spectrum disorder; TD, typically developing children; DR, dark room: EO, eyes open; K-ABC, Kaufman Assessment Battery for Children; SRS, Social Responsiveness scale; MPS, Mental Processing scale; MPI, Mental Processing index.

Asterisks indicate significance

### 3.2 Effect of experimental condition on PAF

Separate linear mixed-effects regression analyses were conducted to predict PAF across all brain regions (cingulate, frontal, occipital, parietal, and temporal) in both the left and right hemispheres. The models included fixed effects for diagnosis (ASD vs. TD), experimental condition (DR vs. EO), their interaction term, age, and sex, with a random intercept for each participant to account for within-subject correlations.

Age was a significant positive predictor of PAF across all regions (left cingulate: z = 5.58, p < 0.0001; left frontal: z = 4.05, p < 0.0001; left occipital: z = 5.74, p < 0.0001; left parietal: z = 7.16, p < 0.0001; left temporal: z = 5.83, p < 0.0001; right cingulate: z = 5.97, p < 0.0001; right frontal: z = 4.43, p = 0.0001; right occipital: z = 4.28, p < 0.001; right parietal: z = 6.34, p < 0.0001; right temporal: z = 4.41, p < 0.0001), indicating that older children tended to have higher PAF values.

As previous studies have suggested that the relationship between age and PAF may differ across various age ranges in both TD and ASD populations, we conducted an exploratory analysis to examine the relationship between age and PAF separately in each group. The association between age and PAF remained significant in both groups across most regions. The results are presented in **Supplementary Table 3**.

In the left and right occipital regions, we observed a significant main effect of experimental condition (z = 3.64, p = 0.0003; and z = 3.50, p = 0.0005, respectively), indicating a significant increase in PAF from DR to EO in the occipital region, observed bilaterally. In the right temporal region, the diagnosis-by-experimental-condition interaction term was significant (z = −2.79, p = 0.0053), while in the left temporal region, this interaction showed a trend toward significance (z = −2.49, p = 0.0126). Detailed results are presented in **Table 2**.

**Table 2:** Effects of diagnosis, experimental condition, and their interaction on peak alpha frequency across cortical regions.

	Coeff.	S.E.	z	*p*	95% CI		
Cingulate (Left)							
Diagnosis (ASD vs. TD)	0.088	0.153	0.57	0.566	-0.212	–	0.389
Experimental condition (DR vs. EO)	0.104	0.107	0.97	0.332	-0.105	–	0.313
Diagnosis * Experimental condition	-0.330	0.163	-2.03	0.043	-0.648	–	-0.011
Age	0.037	0.007	5.58	<0.001*	0.024	–	0.050
Sex	0.265	0.131	2.02	0.043	0.007	–	0.522
Frontal (Left)							
Diagnosis (ASD vs. TD)	-0.037	0.161	-0.23	0.818	-0.352	–	0.279
Experimental condition (DR vs. EO)	0.009	0.083	0.11	0.916	-0.154	–	0.172
Diagnosis * Experimental condition	-0.031	0.128	-0.24	0.811	-0.281	–	0.220
Age	0.031	0.008	4.05	<0.001*	0.015	–	0.046
Sex	0.222	0.148	1.50	0.135	-0.068	–	0.513
Occipital (Left)							
Diagnosis (ASD vs. TD)	-0.036	0.206	-0.17	0.862	-0.440	–	0.368
Experimental condition (DR vs. EO)	0.668	0.183	3.64	<0.001*	0.307	–	1.028
Diagnosis * Experimental condition	-0.331	0.283	-1.17	0.241	-0.884	–	0.223
Age	0.044	0.008	5.74	<0.001*	0.029	–	0.060
Sex	0.386	0.156	2.48	0.013	0.080	–	0.691
Parietal (Left)							
Diagnosis (ASD vs. TD)	-0.100	0.134	-0.75	0.456	-0.362	–	0.163
Experimental condition (DR vs. EO)	0.101	0.093	1.09	0.275	-0.080	–	0.284
Diagnosis * Experimental condition	-0.094	0.145	-0.65	0.517	-0.377	–	0.190
Age	0.041	0.006	7.16	<0.001*	0.020	–	0.052
Sex	0.363	0.113	3.21	0.001*	0.141	–	0.585
Temporal (Left)							
Diagnosis (ASD vs. TD)	0.030	0.178	0.17	0.866	-0.319	–	0.379
Experimental condition (DR vs. EO)	0.309	0.135	2.28	0.023	0.043	–	0.574
Diagnosis * Experimental condition	-0.522	0.209	-2.49	0.013	-0.931	–	-0.112
Age	0.043	0.007	5.83	<0.001*	0.028	–	0.057
Sex	0.345	0.140	2.39	0.017	0.061	–	0.629
Cingulate (Right)							
Diagnosis (ASD vs. TD)	0.079	0.137	0.57	0.566	-0.190	–	0.348
Experimental condition (DR vs. EO)	0.147	0.078	1.90	0.058	-0.004	–	0.299
Diagnosis * Experimental condition	-0.252	0.119	-2.11	0.035	-0.485	–	-0.018
Age	0.038	0.006	5.97	<0.001*	0.025	–	0.051
Sex	0.214	0.125	1.71	0.087	-0.031	–	0.460
Frontal (Right)							
Diagnosis (ASD vs. TD)	0.053	0.160	0.33	0.740	-0.260	–	0.366
Experimental condition (DR vs. EO)	0.109	0.106	1.03	0.303	-0.098	–	0.317
Diagnosis * Experimental condition	-0.178	0.162	-1.10	0.269	-0.494	–	0.138
Age	0.031	0.007	4.43	<0.001*	0.017	–	0.045
Sex	0.182	0.139	1.31	0.190	-0.090	–	0.455
Occipital (Right)							
Diagnosis (ASD vs. TD)	-0.068	0.229	-0.30	0.766	-0.517	–	0.381
Experimental condition (DR vs. EO)	0.608	0.174	3.50	<0.001*	0.268	–	0.949
Diagnosis * Experimental condition	-0.355	0.264	-1.34	0.180	-0.873	–	0.164
Age	0.042	0.010	4.28	<0.001*	0.022	–	0.061
Sex	0.584	0.192	3.04	0.002	0.207	–	0.961
Parietal (Right)							
Diagnosis (ASD vs. TD)	-0.062	0.135	-0.46	0.645	-0.326	–	0.202
Experimental condition (DR vs. EO)	0.063	0.097	0.65	0.515	-0.126	–	0.252
Diagnosis * Experimental condition	-0.074	0.147	-0.50	0.615	-0.362	–	0.214
Age	0.037	0.006	6.34	<0.001*	0.025	–	0.048
Sex	0.421	0.114	3.70	<0.001*	0.197	–	0.645
Temporal (Right)							
Diagnosis (ASD vs. TD)	0.471	0.195	2.42	0.016	0.089	–	0.853
Experimental condition (DR vs. EO)	0.387	0.155	2.50	0.012	0.083	–	0.690
Diagnosis * Experimental condition	-0.662	0.238	-2.79	0.005*	-1.120	–	-0.196
Age	0.034	0.008	4.41	<0.001*	0.018	–	0.049
Sex	0.167	0.153	1.09	0.276	-0.133	–	0.468

ASD, autism spectrum disorder; TD, typically developing; DR, dark room; EO, eyes open; 95% CI, 95% confidence interval; Coeff., coefficient; S.E., standard error.

Asterisks indicate significance.

**Figure 3:**
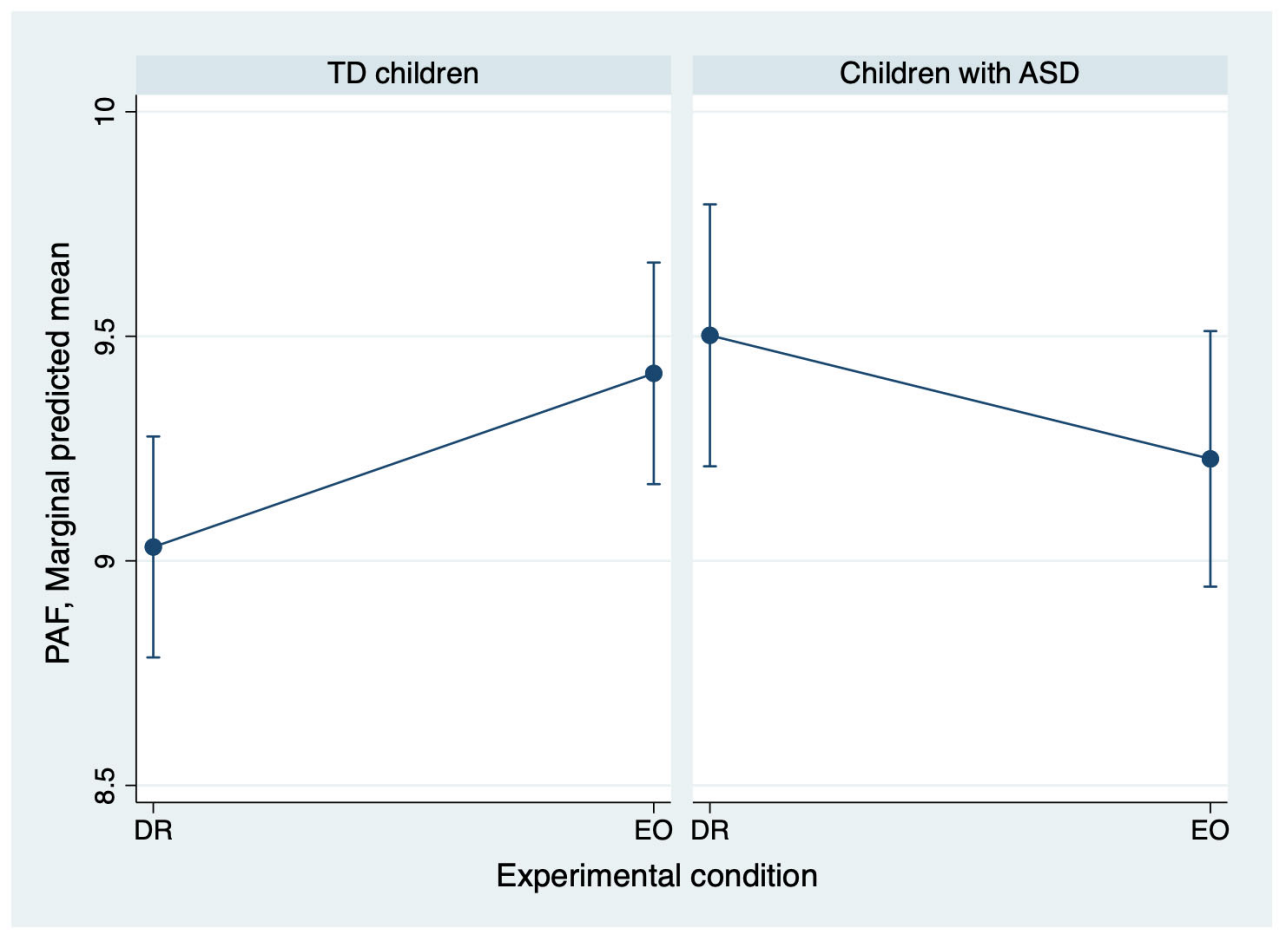
Adjusted mean PAF in the right temporal region for ASD and TD groups. The means were estimated from a linear mixed-effects model that included fixed effects for experimental condition, diagnosis, their interaction, age, and sex, with a random intercept for each participant. Error bars represent the standard error of these adjusted means. The figure illustrates the diagnosis-by-experimental-condition interaction: the ASD group shows a trend toward decreased PAF, while the TD group show a significant increase in PAF from DR to EO. ASD, autism spectrum disorder; PAF, peak alpha frequency; EO, eyes open; DR, dark room; TD children, typically developing children.

To clarify these interactions further, a within-group analysis was conducted. For the right temporal region, TD children showed a significant main effect of experimental condition (z = 2.39, p = 0.017), indicating higher PAF under the EO condition compared with the DR condition, whereas children with ASD showed a nonsignificant trend in the opposite direction (z = −1.63, p = 0.103). Although the interaction did not reach significance, a similar pattern was observed in the left temporal region: TD children again showed a significant main effect of experimental condition (z = 2.67, p = 0.0076), whereas the results for ASD children did not reach significance (z = −1.23, p = 0.2203). Detailed results from these subgroup analyses are presented in **Figure 3** and **Table 3**.

**Table 3:** Within-group effects of experimental condition on peak alpha frequency in temporal regions.

	Coeff.	S.E.	z	*p*	95% CI		
Temporal (Right)							
TD							
Experimental condition (DR vs. EO)	0.387	0.162	2.390	0.017*	0.069	–	0.705
Age	0.024	0.008	2.910	0.004*	0.008	–	0.041
Sex	-0.119	0.163	-0.730	0.467	-0.438	–	0.201
ASD							
Experimental condition (DR vs. EO)	-0.261	0.160	-1.630	0.103	-0.574	–	0.053
Age	0.044	0.013	3.390	<0.001*	0.019	–	0.070
Sex	0.557	0.263	2.120	0.034	0.042	–	1.072
Temporal (Left)							
TD							
Experimental condition (DR vs. EO)	0.309	0.116	2.670	0.008*	0.082	–	0.536
Age	0.036	0.006	6.130	<0.001*	0.025	–	0.048
Sex	0.079	0.116	0.680	0.495	-0.149	–	0.307
ASD							
Experimental condition (DR vs. EO)	-0.213	0.173	-1.230	0.220	-0.553	–	0.127
Age	0.048	0.015	3.180	0.002*	0.018	–	0.077
Sex	0.708	0.299	2.370	0.018	0.122	–	1.294

ASD, autism spectrum disorder; TD, typically developing; DR, dark room; EO, eyes open; 95% CI, 95% confidence interval; Coeff., coefficient; S.E., standard error.

Asterisks indicate significance.

### 3.3 Relationship between EO-induced changes and baseline PAF

To determine whether EO-induced changes depended on baseline DR PAF values, difference in PAF between EO and DR conditions was calculated, and baseline DR PAF was used to predict this difference. Separate linear regression analyses were conducted for each brain region, including fixed effects for diagnosis (ASD vs. TD), baseline PAF, age, and sex. Heteroscedasticity-robust standard errors were applied owing to violations of the homoscedasticity assumption in some models [37].

We also tested models including an interaction term between diagnosis and baseline PAF to assess group differences in the predictive relationship. These interaction terms were not statistically significant in any region (see **Supplementary Table 4**). Therefore, we report results from the more parsimonious models without interaction terms for clarity and interpretability.

Baseline PAF significantly predicted EO-induced changes in all but the right occipital region (left cingulate: t (46) = –3.78, p = 0.0005; left frontal: t (45) = –3.78, p = 0.0005; left occipital: t (38) = –4.10, p = 0.0002; left parietal: t (44) = –2.91, p = 0.0057; left temporal: t (45) = –3.23, p = 0.0023; right cingulate: t(45) = –5.59, p < 0.0001; right frontal: t(46) = –5.90, p < 0.0001; right parietal(46): t = –3.88, p = 0.0003; right temporal(42): t = –3.35, p = 0.0017). In these regions, higher baseline PAF was associated with a smaller increase from DR to EO.

Age also had a significant effect in most regions (left cingulate: t(46) = 2.83, p = 0.0068; left occipital(38): t = 5.35, p < 0.0001; left temporal: t(45) = 2.96, p = 0.0049; right cingulate: t(45) = 5.70, p < 0.0001; right frontal: t(46) = 3.68, p = 0.0006; right parietal: t(46) = 3.71, p = 0.0006; right temporal: t(42) = 2.92, p = 0.0056), indicating that older children tended to show a larger increase in PAF from DR to EO. Detailed results are presented in **Table 4**.

**Table 4:** Baseline peak alpha frequency and age as predictors of eyes-open-induced peak alpha frequency changes across cortical regions.

	Coeff.	Robust S.E.	t	*p*	95% CI		
Vs. EO-induced PAF changes (EO-DR)							
Cingulate (Left)							
PAF in DR condition	-0.588	0.156	-3.780	<0.001*	-0.902	–	-0.275
Diagnosis	-0.274	0.136	-2.010	0.050	-0.548	–	-0.000
Age	0.025	0.009	2.830	0.007*	0.007	–	0.044
Sex	0.079	0.132	0.600	0.553	-0.187	–	0.344
Frontal (Left)							
PAF in DR condition	-0.446	0.118	-3.780	0.001*	-0.684	–	-0.209
Diagnosis	-0.089	0.103	-0.860	0.395	-0.296	–	0.119
Age	0.014	0.008	1.720	0.092	-0.002	–	0.030
Sex	0.060	0.105	0.570	0.568	-0.151	–	0.272
Occipital (Left)							
PAF in DR condition	-0.948	0.232	-4.100	<0.001*	-1.417	–	-0.480
Diagnosis	-0.194	0.197	-0.990	0.330	-0.592	–	0.204
Age	0.051	0.010	5.350	<0.001*	0.032	–	0.071
Sex	0.088	0.185	0.480	0.635	-0.286	–	0.463
Parietal (Left)							
PAF in DR condition	-0.597	0.205	-2.910	0.006*	-1.011	–	-0.183
Diagnosis	-0.110	0.132	-0.830	0.412	-0.377	–	0.157
Age	0.031	0.013	2.390	0.021	0.005	–	0.057
Sex	0.224	0.173	1.290	0.203	-0.126	–	0.574
Temporal (Left)							
PAF in DR condition	-0.624	0.193	-3.230	0.002*	-1.013	–	-0.235
Diagnosis	-0.509	0.198	-2.580	0.013	-0.907	–	-0.111
Age	0.031	0.011	2.960	0.005*	0.010	–	0.053
Sex	0.245	0.163	1.510	0.139	-0.083	–	0.573
Cingulate (Right)							
PAF in DR condition	-0.514	0.092	-5.590	<0.001*	-0.699	–	-0.329
Diagnosis	-0.217	0.090	-2.400	0.021	-0.399	–	-0.035
Age	0.027	0.005	5.700	<0.001*	0.018	–	0.037
Sex	0.026	0.093	0.280	0.782	-0.161	–	0.212
Frontal (Right)							
PAF in DR condition	-0.657	0.111	-5.900	<0.001*	-0.882	–	-0.433
Diagnosis	-0.140	0.109	-1.290	0.204	-0.359	–	0.079
Age	0.023	0.006	3.680	0.001*	0.011	–	0.036
Sex	0.056	0.114	0.490	0.625	-0.173	–	0.284
Occipital (Right)							
PAF in DR condition	-0.392	0.251	-1.560	0.127	-0.899	–	0.116
Diagnosis	-0.342	0.251	-1.370	0.180	-0.850	–	0.166
Age	0.029	0.016	1.740	0.090	-0.005	–	0.062
Sex	0.374	0.290	1.290	0.206	-0.214	–	0.961
Parietal (Right)							
PAF in DR condition	-0.613	0.158	-3.880	<0.001*	-0.932		-0.295
Diagnosis	-0.108	0.123	-0.880	0.384	-0.355	–	0.139
Age	0.031	0.008	3.710	0.001*	0.014	–	0.048
Sex	0.128	0.141	0.910	0.368	-0.156	–	0.412
Temporal (Right)							
PAF in DR condition	-0.734	0.219	-3.350	0.002*	-1.176	–	-0.291
Diagnosis	-0.264	0.240	-1.100	0.277	-0.748	–	0.220
Age	0.028	0.010	2.920	0.006*	0.009	–	0.048
Sex	0.010	0.189	0.050	0.958	-0.372	–	0.392

ASD, autism spectrum disorder; TD, typically developing children; DR, dark room; EO, eyes open; 95% CI, 95% confidence interval; Coeff., coefficient; S.E., standard error.

Asterisks indicate significance.

### 3.4 Relationship between EO-induced changes and autistic traits

Given the significant diagnosis-by-experimental condition interactions in the models predicting PAF in the right temporal region, we investigated whether autistic traits were associated with EO-induced changes in PAF specifically in this area. Additionally, because a similar interaction trend was observed in the left temporal region, the same analysis was conducted.

We calculated the difference in PAF between the two experimental conditions (EO minus DR) for each participant and used linear regression to predict raw total SRS scores based on this difference, age, and sex. Diagnostic checks indicated that the residuals from the initial model violated the assumption of normality; therefore, we log-transformed the dependent variable (raw total SRS scores) to improve the residual distribution.

**Figure 4:**
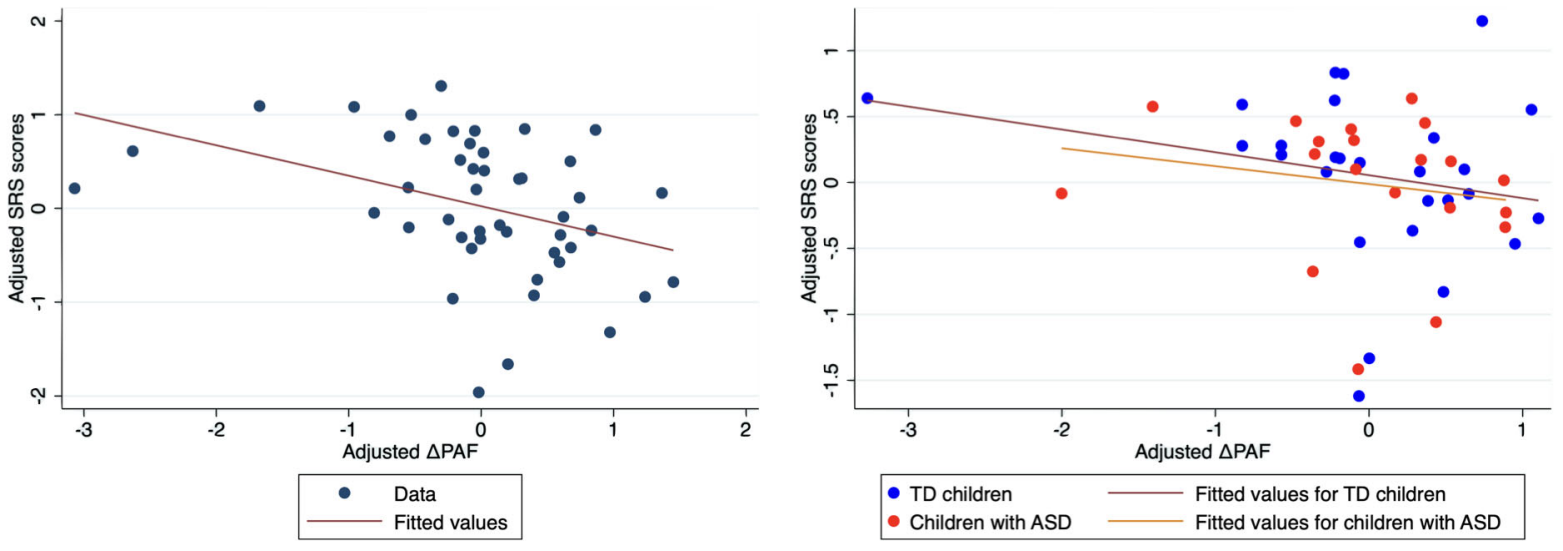
Scatter plots of adjusted raw SRS scores versus adjusted change in PAF in the right temporal region. Left panel. To visualize the results of the multiple regression models, we adjusted both the log-transformed raw SRS scores and change in PAF (DPAF) for age and sex. First, we regressed logtransformed raw SRS scores on age and sex to obtain residuals (adjusted SRS scores). Second, we regressed change in PAF on age and sex to obtain residuals (adjusted DPAF). Third, we created scatter plots of adjusted SRS scores versus adjusted DPAF, along with regression lines representing the relationships. Right panel. Group‐specific associations between social responsiveness and DPAF. First, we regressed log-transformed raw SRS scores on age and sex separately for each group to obtain residuals (adjusted SRS scores). Second, we regressed change in PAF on age and sex to obtain residuals (adjusted DPAF) for each group. Third, we created scatter plots of adjusted SRS scores versus adjusted DPAF for each group, along with regression lines representing the relationships. ASD, autism spectrum disorder; SRS, Social Responsiveness Scale; PAF, peak alpha frequency; TD, typically developing children.

We found a significant effect of the PAF difference in both temporal regions (right temporal: t(43) = –2.88, p = 0.0062; left temporal: t(46) = –4.05, p = 0.0002), indicating that individuals with more pronounced autistic traits exhibited smaller increases in PAF from DR to EO. Detailed results are shown in **Table 5** and **Figure 4**.

**Table 5:** Associations between eyes-open-induced temporal peak alpha frequency changes and autistic traits.

	Coeff.	Robust S.E.	t	*p*	95% CI		
Vs. Log-transformed raw total SRS scores							
Temporal (Right)							
Difference in PAF (EO-DR)	-0.324	0.113	-2.880	0.006*	-0.552	–	-0.097
Age	0.017	0.011	1.610	0.114	-0.004	–	0.039
Sex	0.205	0.211	0.970	0.337	-0.220	–	0.630
Temporal (Left)							
Difference in PAF (EO-DR)	-0.447	0.110	-4.050	<0.001*	-0.669	–	-0.225
Age	0.013	0.011	1.170	0.249	-0.009	–	0.035
Sex	0.252	0.201	1.250	0.217	-0.153	–	0.657

ASD, autism spectrum disorder; TD, typically developing children; SRS, social responsiveness scale; DR, dark room; EO, eyes open; 95% CI, 95% confidence interval; Coeff., coefficient; S.E., standard error.

Asterisks indicate significance.

## 4 Discussion

This study examined whether children with ASD differ from their TD peers in PAF reactivity across two conditions: a DR resting state and an EO condition involving silent, self-selected video viewing. We also explored whether these differences relate to the severity of autistic traits. In the DR condition, children lay supine in a DR while fixating on a central cross, approximating classical resting-state protocols. In the EO condition, they remained supine while watching a silent, personally chosen video projected onto a screen. Specifically, we asked (a) whether PAF shifts from DR to EO differ between diagnostic groups and (b) whether these shifts correlate with SRS scores. By focusing on this transition from minimal to rich visual input, we aimed to clarify how visual stimulation modulates alpha oscillations in children with neurodevelopmental conditions. Our analyses revealed that older children, regardless of diagnosis, generally showed higher PAF values across multiple brain regions, a finding that aligns with those of previous developmental studies on alpha frequency maturation. In the occipital region, both the ASD and TD groups displayed elevated PAF under the EO condition, suggesting that alpha activity in primary visual areas is responsive to increased visual input in a relatively uniform manner. However, in the temporal regions, a significant (or trend-level) diagnosis-by-condition interaction was found: TD children consistently showed higher PAF under the EO condition relative to the DR condition, whereas children with ASD did not. Furthermore, baseline PAF during DR predicted the magnitude of PAF change induced by EO, with higher baseline values generally linked to a smaller increase (or decrease) in alpha frequency. Finally, we found that children with more pronounced autistic traits exhibited less EO-related enhancement of PAF in temporal areas, suggesting that atypical alpha reactivity to visual stimuli in ASD may be associated with the severity of social-communicative difficulties.

A key finding of this study was that older children consistently showed higher PAF values than younger children, a pattern observed across multiple cortical regions. This aligns with prior findings suggesting that PAF increases with age in TD individuals (29, 32, 33, 38). In our exploratory analyses, we found that the association between age and PAF remained significant in both groups across most regions (**Supplementary Table 3**). While several studies have reported age-related increases in PAF in the TD group and the group with ASD, including Shen et al. (29), importantly, in children (16, 29, 32, 33) or adolescents (38) with ASD, the age–PAF relationship has not consistently reached statistical significance. A negative association has even been reported in adults (30). Of particular relevance, two of these studies (29, 33) included children with similar age ranges to ours―7.5 ± 0.8 years (TD) vs. 7.8 ± 0.8 years (ASD) in Shen et al. (29) and 5.96 ± 2.2 years (TD) vs. 5.76 ± 2.0 years (ASD) in Dickinson et al. (33)―but did not show the same positive association. Methodological differences may explain these discrepancies. For example, Shen et al. (29) derived a single, representative “peak alpha” from nine posterior regions, whereas Dickinson et al. (33) calculated an average PAF over just six electrode sites, merging pairs (F3–F4, C3–C4, O1–O2). In contrast, we estimated PAF for ten source-localized regions separately, providing a more fine-grained measure. Moreover, medication usage in some participants of the Dickinson et al. (33) study may have influenced alpha dynamics. Indeed, Kameya et al. (37), who used a methodology identical to ours and excluded participants using medication, found a similarly positive association between age and PAF, although only in the right and left cingulate regions, possibly owing to the smaller sample size (24 TD children aged 69.6 ± 9.0 months; 19 children with ASD aged 72.5 ± 7.5 months; data collected only under EO condition with minimal visual stimulation). Overall, our results extend prior findings by demonstrating, within a single cohort, that the positive correlation between age and PAF is independent of the experimental condition in children (mean age ~6 years for both TD and ASD). Our findings also underscore how the choice of analytical methods and participant inclusion criteria (such as medication use) might shape our interpretation of the developmental trajectory of PAF. These observations highlight the importance of considering age-related changes when examining PAF in patients with neurodevelopmental disorders.

Another noteworthy observation from our study was the significant increase in PAF from DR to EO in the occipital region, which was observed bilaterally. At first glance, this finding may appear to contrast with the recent meta-analysis by Freschl et al. (67), who reported no effect of EO versus EC on resting-state occipital PAF in individuals aged 0–18 years of age. However, Freschl et al. collapsed all eyes-open methodologies into a single category—combining data acquired under DR, lit-room, and visually stimulating conditions—due to limited sample sizes within each subgroup. This grouping might have masked meaningful differences across conditions. Supporting this view, Edgar et al. (68) examined children aged 6.9–12.6 and found that PAF measured during an EO condition in a DR was comparable to that measured during EC. Our results extend those of Edgar et al. by demonstrating that an EO condition involving robust visual stimulation systematically produces higher PAF compared to the DR condition. Notably, we observed a main effect of experimental condition in the occipital cortex without a significant diagnosis-by-condition interaction, suggesting that children in both ASD and TD groups exhibit a similar degree of alpha reactivity to increased visual stimulation in this region.

Turning to the temporal region, we observed a notable diagnosis-by-condition interaction: TD children consistently showed higher PAF under the EO condition compared the DR condition, whereas children with ASD did not. This pattern contrasts with that of the occipital region, where PAF reactivity to increased visual input appeared largely similar between the groups. Recent research suggests that alpha oscillations in the temporal region play an important role in creativity (69, 70) and in actively inhibiting distractions during working memory tasks (71). Interestingly, individuals with ASD often exhibit atypical creativity profiles (72) and reduced working memory capacity (73). Therefore, the lack of clear temporal PAF reactivity among children with ASD may reflect atypical neural mechanisms in regions responsible for these functions, suggesting a link between temporal PAF reactivity and autistic traits. Alternatively, it might indicate a reduced capacity to modulate alpha frequency in response to more complex or sustained visual inputs, a capacity that appears to be relatively preserved in TD children. Consequently, it remains unclear whether EO-related enhancements in temporal PAF are directly related to autistic traits.

To the best of our knowledge, this study is the first to show that children with more pronounced autistic traits, as measured by the SRS, exhibit reduced EO-related increase (or decrease) in temporal PAF. This finding suggests that atypical alpha reactivity to visual stimuli in ASD may be related to the severity of social-communicative difficulties. The temporal cortex has long been implicated in social cognition (74), perception, and interaction (72, 75), processes that depend heavily on the integration of both sensory input and higher-order cognitive functions. Diminished alpha reactivity in these regions could signify reduced neural flexibility, potentially limiting the brain’s ability to adapt to dynamic visual environments that are crucial for social engagement. Nevertheless, given our relatively small sample size and the limited variability in participant characteristics (e.g., absence of intellectual disability and a narrow age range), it would be premature to draw definitive conclusions. Further research with larger samples and a broader spectrum of participants is necessary to determine whether these findings can be generalized.

Finally, we found that baseline PAF under the DR condition predicted the magnitude of subsequent PAF change in response to EO, such that higher baseline values were generally associated with a smaller (or even negative) shift in alpha frequency. To the best of our knowledge, no previous studies have directly examined this relationship. One possible explanation is that alpha oscillations may exhibit a “ceiling effect,” whereby children whose resting alpha frequency is already relatively high have limited capacity for further enhancement when exposed to increased visual input. Conversely, those with lower baseline alpha frequency may show more flexibility or “headroom” for neurophysiological adjustment, resulting in a larger EO-related increase. These findings highlight the importance of considering initial resting-state parameters when interpreting alpha reactivity, as baseline PAF likely constrains subsequent changes.

This study has several limitations. First, the modest sample size may have limited our ability to detect subtle group differences in PAF. Future studies with larger and more diverse samples are required to enhance the generalizability of our findings (76). Second, our participants comprised a narrow age range (approximately 5–10 years) and excluded individuals with intellectual disabilities and those using medication. Thus, our findings may not be generalizable to all children with ASD, particularly those with intellectual disabilities, with comorbid psychiatric conditions, or at different developmental stages. Third, although the use of individualized video stimuli in the EO condition helped reduce stress and minimize motion artifacts—especially critical in pediatric populations—it introduced variability in visual input across participants, which could have influenced neural responses. The children selected video programs; however, the specific content varied widely, with almost no overlap in chosen videos. This diversity made it infeasible to categorize or quantitatively compare stimulus properties between the group with ASD and the TD group. Although both groups had access to the same set of age-appropriate video options, we cannot rule out the possibility that differences in visual complexity, motion dynamics, or social content influenced alpha reactivity. Therefore, caution is warranted when interpreting group differences in the EO condition, and future studies may benefit from using a standardized set of stimuli to isolate diagnosis-related effects better. Fourth, another important limitation concerns the possibility that group differences in PAF reactivity may reflect behavioral differences during the scanning session. Although participants were instructed to keep their eyes open and watch a preferred silent video, we could not directly monitor attentional engagement or compliance (e.g., eye closure or gaze aversion) during MEG acquisition. Given that alpha oscillations are sensitive to eye state and visual input, reduced PAF reactivity in children with ASD may partly reflect variability in task engagement or attention to the video rather than solely underly neural differences. Future studies incorporating real-time behavioral monitoring (e.g., eye tracking or video surveillance) would help clarify the contribution of attentional factors to alpha modulation. Fifth, owing to the cross-sectional nature of our design, we could not determine the longitudinal stability of our findings or infer causality between PAF reactivity and autistic traits. Future longitudinal studies with larger and more diverse samples are required to validate and extend our results. Sixth, a limitation concerns the reliability of PAF estimation at the individual level. Due to the large data volume (68 PSDs per participant × 51 participants), we could not include individual power spectra. Instead, we assessed plausibility by calculating the proportion of predicted PAF values falling within the physiologically expected range (7–13 Hz). These proportions were generally high in the TD group (70.7–97.9%) but more variable and often lower in the group with ASD (42.5–69.0%), suggesting reduced robustness in some individuals. While this may reflect meaningful neurophysiological differences, it is more likely due to greater signal variability, artifacts, or atypical alpha topography. To mitigate this concern, we averaged source-level data into 10 broader anatomical regions to improve stability. This likely enhanced alpha peak detection, especially in participants with noisier data. Nonetheless, findings in regions with lower detectability should be interpreted with caution. Although we retained this PAF calculation method to maintain consistency with previous studies using the same approach (30, 33, 34, 37–39), future research may benefit from refined preprocessing pipelines (e.g., using the publicly available FOOOF package: https://fooof-tools.github.io/fooof/) to improve PAF detectability in clinical populations. Seventh, related to the issue above, we also acknowledge limitations in evaluating the spectral fit quality. While we reported R² values to quantify how well the modeled spectrum fit the observed PSD, these values reflect the overall fit, including both periodic (e.g., alpha peak) and aperiodic components. Thus, lower R² values do not necessarily indicate inaccurate estimation of the peak alpha frequency; instead, they may arise from imperfect modeling of the aperiodic background. Given that our primary objective was to determine the peak alpha location, we do not consider high R² values to be essential for the validity of our conclusions. To further assess the plausibility of peak detection, we visually inspected the PSDs and fitted curves across all regions. This evaluation was independently performed by two authors (T.H. and M.S.). Although the large number of plots (68 per participant × 51 participants) precluded inclusion in the manuscript, they are available upon request from the corresponding author. This visual confirmation process complemented the quantitative analysis and helped ensure the reliability of detected peaks.

In summary, our findings provide new insights into how children with ASD and their TD peers differ in PAF reactivity under DR and robust visual input conditions. First, we observed that older children, regardless of diagnosis, tended to have higher PAF values, underscoring the importance of considering developmental trajectories when interpreting PAF. Second, while both groups exhibited a similar PAF increase in occipital regions during video viewing, the temporal regions showed a diagnosis-by-condition interaction: TD children consistently displayed elevated PAF during the video condition, whereas children with ASD did not. This group difference was further moderated by autistic traits, with more pronounced traits associated with a reduced—or even negative—shift in temporal PAF in response to visual input. Lastly, baseline PAF under the DR condition predicted the degree of modulation during EO silent video viewing, suggesting that individual differences in DR alpha may constrain dynamic reactivity to visual stimulation. These findings highlight temporal PAF reactivity as a potential marker of altered sensory processing in ASD. Future research—ideally longitudinal—should examine these patterns in larger and more diverse ASD samples, including individuals with intellectual disabilities and psychiatric comorbidities, to enhance generalizability. Such studies should also employ a standardized set of stimuli to better isolate diagnosis-related effects on PAF reactivity to visual input.

## Data Availability

The raw data supporting the conclusions of this article will be made available by the authors, without undue reservation.
