# Supplementary material for: State-dependent changes in peak alpha frequency during visual engagement in children with and without autism spectrum disorder
Source: Front Psychiatry. 2025 Oct 6;16:1634384. doi: 10.3389/fpsyt.2025.1634384 (PMC12536023; doi:10.3389/fpsyt.2025.1634384)
Supplement: Supplementary file 1 [file Table1.docx]

Supplementary Material

**Supplementary Table 1. Regional detectability of PAF (proportion of estimates within 7–13 Hz), by macro-region and condition.**

|  | ASD |  | TD |  |  |
| --- | --- | --- | --- | --- | --- |
|  | EO | DR | EO | DR |  |
| Left cingulate | 65.50% | 67.60% | 89.70% | 93.80% |  |
| Left frontal | 58.60% | 64.30% | 84.60% | 86.50% |  |
| Left occipital | 55.20% | 65.50% | 70.70% | 92.20% |  |
| Left parietal | 62.10% | 68.30% | 91.70% | 97.90% |  |
| Left temporal | 42.50% | 58.20% | 72.40% | 87.00% |  |
| Right cingulate | 64.80% | 69.00% | 90.30% | 92.40% |  |
| Right frontal | 61.10% | 62.70% | 86.80% | 85.90% |  |
| Right occipital | 52.60% | 63.80% | 73.30% | 90.50% |  |
| Right parietal | 66.90% | 68.30% | 91.00% | 93.10% |  |
| Right temporal | 51.70% | 56.30% | 72.80% | 78.90% |  |

ASD, autism spectrum disorder; TD, typically developing; DR, dark room; EO, eyes open.

**Supplementary Table 2.** Mean R² values between modeled and observed power spectral densities for each group, region, and condition.

|  | ASD |  | TD |  |
| --- | --- | --- | --- | --- |
|  | EO | DR | EO | DR |
| Left cingulate | 0.585 | 0.637 | 0.557 | 0.638 |
| Left frontal | 0.602 | 0.616 | 0.581 | 0.605 |
| Left occipital | 0.464 | 0.766 | 0.408 | 0.714 |
| Left parietal | 0.661 | 0.697 | 0.618 | 0.689 |
| Left temporal | 0.467 | 0.573 | 0.390 | 0.530 |
| Right cingulate | 0.568 | 0.612 | 0.562 | 0.622 |
| Right frontal | 0.590 | 0.605 | 0.545 | 0.588 |
| Right occipital | 0.474 | 0.752 | 0.419 | 0.708 |
| Right parietal | 0.692 | 0.697 | 0.642 | 0.655 |
| Right temporal | 0.449 | 0.556 | 0.385 | 0.541 |

ASD, autism spectrum disorder; TD, typically developing; DR, dark room; EO, eyes open.

**Supplementary Table 3. Associations between age and peak alpha frequency in groups of autism spectrum disorder and typically developing children across cortical regions.**

|  | **Coeff.** | **S.E.** | **z** | ***p*** | **95% CI** | | |
| --- | --- | --- | --- | --- | --- | --- | --- |
| **TD** |  |  |  |  |  |  |  |
| **Cingulate (Left)** |  |  |  |  |  |  |  |
| Experimental condition (DR vs. EO) | 0.104 | 0.120 | 0.860 | 0.390 | -0.132 | - | 0.340 |
| Age | 0.025 | 0.007 | 3.640 | <0.001 | 0.012 | - | 0.039 |
| Sex | 0.179 | 0.137 | 1.310 | 0.191 | -0.089 | - | 0.447 |
|  |  |  |  |  |  |  |  |
| **ASD** |  |  |  |  |  |  |  |
| **Cingulate (Left)** |  |  |  |  |  |  |  |
| Experimental condition (DR vs. EO) | -0.226 | 0.098 | -2.300 | 0.021 | -0.419 | - | -0.034 |
| Age | 0.053 | 0.012 | 4.530 | <0.001 | 0.030 | - | 0.077 |
| Sex | 0.325 | 0.232 | 1.400 | 0.162 | -0.130 | - | 0.779 |
|  |  |  |  |  |  |  |  |
| **TD** |  |  |  |  |  |  |  |
| **Frontal (Left)** |  |  |  |  |  |  |  |
| Experimental condition (DR vs. EO) | 0.009 | 0.089 | 0.100 | 0.921 | -0.165 | - | 0.183 |
| Age | 0.019 | 0.008 | 2.390 | 0.017 | 0.003 | - | 0.035 |
| Sex | -0.007 | 0.158 | -0.040 | 0.964 | -0.317 | - | 0.302 |
|  |  |  |  |  |  |  |  |
| **ASD** |  |  |  |  |  |  |  |
| **Frontal (Left)** |  |  |  |  |  |  |  |
| Experimental condition (DR vs. EO) | -0.033 | 0.085 | -0.390 | 0.696 | -0.200 | - | 0.134 |
| Age | 0.046 | 0.013 | 3.470 | 0.001 | 0.020 | - | 0.072 |
| Sex | 0.496 | 0.256 | 1.930 | 0.053 | -0.006 | - | 0.998 |
|  |  |  |  |  |  |  |  |
| **TD** |  |  |  |  |  |  |  |
| **Occipital (Left)** |  |  |  |  |  |  |  |
| Experimental condition (DR vs. EO) | 0.667 | 0.181 | 3.690 | <0.001 | 0.312 | - | 1.021 |
| Age | 0.039 | 0.009 | 4.190 | <0.001 | 0.021 | - | 0.057 |
| Sex | 0.279 | 0.181 | 1.540 | 0.123 | -0.076 | - | 0.634 |
|  |  |  |  |  |  |  |  |
| **ASD** |  |  |  |  |  |  |  |
| **Occipital (Left)** |  |  |  |  |  |  |  |
| Experimental condition (DR vs. EO) | 0.407 | 0.147 | 2.760 | 0.006 | 0.118 | - | 0.696 |
| Age | 0.055 | 0.016 | 3.420 | 0.001 | 0.023 | - | 0.087 |
| Sex | 0.574 | 0.320 | 1.800 | 0.072 | -0.052 | - | 1.201 |
|  |  |  |  |  |  |  |  |
| **TD** |  |  |  |  |  |  |  |
| **Parietal (Left)** |  |  |  |  |  |  |  |
| Experimental condition (DR vs. EO) | 0.101 | 0.105 | 0.970 | 0.332 | -0.104 | - | 0.306 |
| Age | 0.036 | 0.007 | 5.500 | <0.001 | 0.023 | - | 0.049 |
| Sex | 0.347 | 0.130 | 2.670 | 0.008 | 0.093 | - | 0.601 |
|  |  |  |  |  |  |  |  |
| **ASD** |  |  |  |  |  |  |  |
| **Parietal (Left)** |  |  |  |  |  |  |  |
| Experimental condition (DR vs. EO) | 0.028 | 0.083 | 0.340 | 0.732 | -0.134 | - | 0.190 |
| Age | 0.048 | 0.011 | 4.590 | <0.001 | 0.028 | - | 0.069 |
| Sex | 0.347 | 0.208 | 1.670 | 0.095 | -0.060 | - | 0.754 |
|  |  |  |  |  |  |  |  |
| **TD** |  |  |  |  |  |  |  |
| **Temporal (Left)** |  |  |  |  |  |  |  |
| Experimental condition (DR vs. EO) | 0.309 | 0.116 | 2.670 | 0.008 | 0.082 | - | 0.536 |
| Age | 0.036 | 0.006 | 6.130 | <0.001 | 0.025 | - | 0.048 |
| Sex | 0.079 | 0.116 | 0.680 | 0.495 | -0.149 | - | 0.307 |
|  |  |  |  |  |  |  |  |
| **ASD** |  |  |  |  |  |  |  |
| **Temporal (Left)** |  |  |  |  |  |  |  |
| Experimental condition (DR vs. EO) | -0.213 | 0.173 | -1.230 | 0.220 | -0.553 | - | 0.127 |
| Age | 0.048 | 0.015 | 3.180 | 0.002 | 0.018 | - | 0.077 |
| Sex | 0.708 | 0.299 | 2.370 | 0.018 | 0.122 | - | 1.294 |
|  |  |  |  |  |  |  |  |
| **TD** |  |  |  |  |  |  |  |
| **Cingulate (Right)** |  |  |  |  |  |  |  |
| Experimental condition (DR vs. EO) | 0.147 | 0.086 | 1.700 | 0.089 | -0.022 | - | 0.316 |
| Age | 0.030 | 0.007 | 4.140 | <0.001 | 0.016 | - | 0.044 |
| Sex | 0.067 | 0.140 | 0.480 | 0.634 | -0.208 | - | 0.342 |
|  |  |  |  |  |  |  |  |
| **ASD** |  |  |  |  |  |  |  |
| **Cingulate (Right)** |  |  |  |  |  |  |  |
| Experimental condition (DR vs. EO) | -0.102 | 0.074 | -1.370 | 0.170 | -0.248 | - | 0.044 |
| Age | 0.048 | 0.011 | 4.440 | <0.001 | 0.027 | - | 0.069 |
| Sex | 0.384 | 0.213 | 1.810 | 0.071 | -0.033 | - | 0.802 |
|  |  |  |  |  |  |  |  |
| **TD** |  |  |  |  |  |  |  |
| **Frontal (Right)** |  |  |  |  |  |  |  |
| Experimental condition (DR vs. EO) | 0.109 | 0.111 | 0.990 | 0.324 | -0.108 | - | 0.326 |
| Age | 0.021 | 0.008 | 2.600 | 0.009 | 0.005 | - | 0.036 |
| Sex | -0.056 | 0.157 | -0.360 | 0.719 | -0.364 | - | 0.251 |
|  |  |  |  |  |  |  |  |
| **ASD** |  |  |  |  |  |  |  |
| **Frontal (Right)** |  |  |  |  |  |  |  |
| Experimental condition (DR vs. EO) | -0.069 | 0.114 | -0.600 | 0.546 | -0.293 | - | 0.155 |
| Age | 0.043 | 0.011 | 3.820 | <0.001 | 0.021 | - | 0.066 |
| Sex | 0.472 | 0.223 | 2.120 | 0.034 | 0.035 | - | 0.909 |
|  |  |  |  |  |  |  |  |
| **TD** |  |  |  |  |  |  |  |
| **Occipital (Right)** |  |  |  |  |  |  |  |
| Experimental condition (DR vs. EO) | 0.583 | 0.201 | 2.900 | 0.004 | 0.189 | - | 0.978 |
| Age | 0.029 | 0.012 | 2.450 | 0.014 | 0.006 | - | 0.053 |
| Sex | 0.437 | 0.234 | 1.870 | 0.062 | -0.022 | - | 0.896 |
|  |  |  |  |  |  |  |  |
| **ASD** |  |  |  |  |  |  |  |
| **Occipital (Right)** |  |  |  |  |  |  |  |
| Experimental condition (DR vs. EO) | 0.283 | 0.142 | 1.990 | 0.047 | 0.004 | - | 0.562 |
| Age | 0.058 | 0.016 | 3.690 | <0.001 | 0.027 | - | 0.089 |
| Sex | 0.728 | 0.313 | 2.330 | 0.020 | 0.116 | - | 1.341 |
|  |  |  |  |  |  |  |  |
| **TD** |  |  |  |  |  |  |  |
| **Parietal (Right)** |  |  |  |  |  |  |  |
| Experimental condition (DR vs. EO) | 0.063 | 0.102 | 0.620 | 0.536 | -0.136 | - | 0.262 |
| Age | 0.028 | 0.006 | 4.830 | <0.001 | 0.017 | - | 0.039 |
| Sex | 0.330 | 0.113 | 2.920 | 0.004 | 0.109 | - | 0.552 |
|  |  |  |  |  |  |  |  |
| **ASD** |  |  |  |  |  |  |  |
| **Parietal (Right)** |  |  |  |  |  |  |  |
| Experimental condition (DR vs. EO) | -0.011 | 0.103 | -0.110 | 0.914 | -0.213 | - | 0.191 |
| Age | 0.048 | 0.011 | 4.530 | <0.001 | 0.027 | - | 0.069 |
| Sex | 0.504 | 0.211 | 2.390 | 0.017 | 0.091 | - | 0.917 |
|  |  |  |  |  |  |  |  |
| **TD** |  |  |  |  |  |  |  |
| **Temporal (Right)** |  |  |  |  |  |  |  |
| Experimental condition (DR vs. EO) | 0.387 | 0.162 | 2.390 | 0.017 | 0.069 | - | 0.705 |
| Age | 0.024 | 0.008 | 2.910 | 0.004 | 0.008 | - | 0.041 |
| Sex | -0.119 | 0.163 | -0.730 | 0.467 | -0.438 | - | 0.201 |
|  |  |  |  |  |  |  |  |
| **ASD** |  |  |  |  |  |  |  |
| **Temporal (Right)** |  |  |  |  |  |  |  |
| Experimental condition (DR vs. EO) | -0.261 | 0.160 | -1.630 | 0.103 | -0.574 | - | 0.053 |
| Age | 0.044 | 0.013 | 3.390 | 0.001 | 0.019 | - | 0.070 |
| Sex | 0.557 | 0.263 | 2.120 | 0.034 | 0.042 | - | 1.072 |

ASD, autism spectrum disorder; TD, typically developing; DR, dark room; EO, eyes open; 95% CI, 95% confidence interval; Coeff., coefficient; S.E., standard error.

**Supplementary Table 4. Linear regression models including interaction between diagnosis and baseline peak alpha frequency, predicting eyes open–dark room peak alpha frequency difference across brain regions.**

|  | **Coeff.** | **Robust S.E.** | **t** | ***p*** | **95% CI** | | |
| --- | --- | --- | --- | --- | --- | --- | --- |
| **Vs. EO-induced PAF changes (EO-DR)** |  |  |  |  |  |  |  |
| **Cingulate (Left)** |  |  |  |  |  |  |  |
| PAF in DR condition | -0.838 | 0.229 | -3.660 | 0.001 | -1.300 | - | -0.377 |
| Diagnosis | -4.102 | 2.153 | -1.910 | 0.063 | -8.438 | - | 0.233 |
| Diagnosis*Experimental condition | 0.416 | 0.234 | 1.780 | 0.082 | -0.054 | - | 0.886 |
| Age | 0.024 | 0.008 | 3.030 | 0.004 | 0.008 | - | 0.040 |
| Sex | 0.069 | 0.125 | 0.550 | 0.585 | -0.183 | - | 0.321 |
|  |  |  |  |  |  |  |  |
| **Frontal (Left)** |  |  |  |  |  |  |  |
| PAF in DR condition | -0.532 | 0.187 | -2.840 | 0.007 | -0.909 | - | -0.154 |
| Diagnosis | -1.533 | 1.985 | -0.770 | 0.444 | -5.534 | - | 2.468 |
| Diagnosis*Experimental condition | 0.158 | 0.219 | 0.720 | 0.476 | -0.284 | - | 0.600 |
| Age | 0.014 | 0.008 | 1.660 | 0.103 | -0.003 | - | 0.030 |
| Sex | 0.044 | 0.113 | 0.390 | 0.696 | -0.183 | - | 0.271 |
|  |  |  |  |  |  |  |  |
| **Occipital (Left)** |  |  |  |  |  |  |  |
| PAF in DR condition | -1.223 | 0.280 | -4.370 | 0.000 | -1.791 | - | -0.656 |
| Diagnosis | -5.622 | 3.426 | -1.640 | 0.109 | -12.565 | - | 1.320 |
| Diagnosis*Experimental condition | 0.595 | 0.367 | 1.620 | 0.114 | -0.149 | - | 1.338 |
| Age | 0.052 | 0.010 | 5.140 | 0.000 | 0.031 | - | 0.072 |
| Sex | 0.106 | 0.176 | 0.600 | 0.551 | -0.251 | - | 0.463 |
|  |  |  |  |  |  |  |  |
| **Parietal (Left)** |  |  |  |  |  |  |  |
| PAF in DR condition | -0.620 | 0.245 | -2.540 | 0.015 | -1.113 | - | -0.127 |
| Diagnosis | -0.490 | 1.406 | -0.350 | 0.729 | -3.325 | - | 2.345 |
| Diagnosis*Experimental condition | 0.041 | 0.148 | 0.280 | 0.783 | -0.258 | - | 0.340 |
| Age | 0.031 | 0.013 | 2.380 | 0.022 | 0.005 | - | 0.057 |
| Sex | 0.224 | 0.175 | 1.280 | 0.208 | -0.129 | - | 0.578 |
|  |  |  |  |  |  |  |  |
| **Temporal (Left)** |  |  |  |  |  |  |  |
| PAF in DR condition | -0.925 | 0.217 | -4.260 | 0.000 | -1.363 | - | -0.487 |
| Diagnosis | -4.279 | 2.752 | -1.550 | 0.127 | -9.824 | - | 1.267 |
| Diagnosis*Experimental condition | 0.412 | 0.307 | 1.340 | 0.187 | -0.207 | - | 1.030 |
| Age | 0.032 | 0.011 | 3.080 | 0.004 | 0.011 | - | 0.054 |
| Sex | 0.222 | 0.155 | 1.430 | 0.160 | -0.091 | - | 0.534 |
|  |  |  |  |  |  |  |  |
| **Cingulate (Right)** |  |  |  |  |  |  |  |
| PAF in DR condition | -0.560 | 0.111 | -5.050 | 0.000 | -0.783 | - | -0.336 |
| Diagnosis | -0.936 | 1.342 | -0.700 | 0.489 | -3.641 | - | 1.769 |
| Diagnosis*Experimental condition | 0.078 | 0.144 | 0.540 | 0.591 | -0.213 | - | 0.369 |
| Age | 0.027 | 0.005 | 5.740 | 0.000 | 0.017 | - | 0.036 |
| Sex | 0.021 | 0.093 | 0.230 | 0.821 | -0.166 | - | 0.208 |
|  |  |  |  |  |  |  |  |
| **Frontal (Right)** |  |  |  |  |  |  |  |
| PAF in DR condition | -0.745 | 0.152 | -4.920 | 0.000 | -1.050 | - | -0.440 |
| Diagnosis | -1.648 | 1.653 | -1.000 | 0.324 | -4.977 | - | 1.680 |
| Diagnosis*Experimental condition | 0.164 | 0.182 | 0.900 | 0.373 | -0.203 | - | 0.530 |
| Age | 0.022 | 0.006 | 3.860 | 0.000 | 0.011 | - | 0.034 |
| Sex | 0.025 | 0.117 | 0.210 | 0.835 | -0.212 | - | 0.261 |
|  |  |  |  |  |  |  |  |
| **Occipital (Right)** |  |  |  |  |  |  |  |
| PAF in DR condition | -0.600 | 0.407 | -1.470 | 0.149 | -1.425 | - | 0.226 |
| Diagnosis | -3.817 | 4.328 | -0.880 | 0.384 | -12.595 | - | 4.961 |
| Diagnosis*Experimental condition | 0.380 | 0.468 | 0.810 | 0.422 | -0.569 | - | 1.329 |
| Age | 0.027 | 0.017 | 1.570 | 0.126 | -0.008 | - | 0.062 |
| Sex | 0.377 | 0.286 | 1.320 | 0.197 | -0.204 | - | 0.958 |
|  |  |  |  |  |  |  |  |
| **Parietal (Right)** |  |  |  |  |  |  |  |
| PAF in DR condition | -0.819 | 0.180 | -4.540 | 0.000 | -1.182 | - | -0.456 |
| Diagnosis | -2.927 | 1.663 | -1.760 | 0.085 | -6.277 | - | 0.422 |
| Diagnosis*Experimental condition | 0.304 | 0.179 | 1.700 | 0.096 | -0.056 | - | 0.663 |
| Age | 0.031 | 0.008 | 3.700 | 0.001 | 0.014 | - | 0.048 |
| Sex | 0.141 | 0.137 | 1.030 | 0.311 | -0.136 | - | 0.417 |
|  |  |  |  |  |  |  |  |
| **Temporal (Right)** |  |  |  |  |  |  |  |
| PAF in DR condition | -0.988 | 0.443 | -2.230 | 0.031 | -1.882 | - | -0.093 |
| Diagnosis | -3.775 | 4.072 | -0.930 | 0.359 | -12.000 | - | 4.449 |
| Diagnosis*Experimental condition | 0.382 | 0.460 | 0.830 | 0.411 | -0.547 | - | 1.310 |
| Age | 0.028 | 0.009 | 3.000 | 0.005 | 0.009 | - | 0.047 |
| Sex | -0.001 | 0.203 | -0.010 | 0.994 | -0.411 | - | 0.408 |

PAF, peak alpha frequency; DR, dark room; EO, eyes open; 95% CI, 95% confidence interval; Coeff., coefficient; S.E., standard error.
